# Strengthening the Healthy Start Workforce: A Mixed-Methods Study to Understand the Roles of Community Health Workers in Healthy Start and Inform the Development of a Standardized Training Program

**DOI:** 10.1007/s10995-017-2377-x

**Published:** 2017-11-18

**Authors:** Katherine Rachel DeAngelis, Katelyn Flaherty Doré, Deborah Dean, Paul Osterman

**Affiliations:** 1 Healthy Start EPIC Center, JSI Research & Training Institute, Inc., 44 Farnsworth Street, Boston, MA 02210 USA; 2 MIT Sloan School of Management, 100 Main Street, Cambridge, MA 02139 USA

**Keywords:** Healthy start, Community health worker, Infant mortality, Maternal health, social determinants of health

## Abstract

*Introduction* Healthy Start (HS) is dedicated to preventing infant mortality, improving birth outcomes, and reducing disparities in maternal and infant health. In 2014, the HS program was reenvisioned and standardization of services and workforce development were prioritized. This study examined how HS community health workers (CHW), as critical members of the workforce, serve families and communities in order to inform the development of a CHW training program to advance program goals. *Methods* In 2015, an online organizational survey of all 100 HS programs was conducted. Ninety-three sites (93%) responded. Three discussion groups were subsequently conducted with HS CHWs (n = 21) and two discussion groups with HS CHW trainers/supervisors (n = 14). *Results* Most (91%) respondent HS programs employed CHWs. Survey respondents ranked health education (90%), assessing participant needs (85%), outreach/recruitment (85%), and connecting participants to services (85%) as the most central roles to the CHW’s job. Survey findings indicated large variation in CHW training, both in the amount and content provided. Discussion group findings provided further examples of the knowledge and skills required by HS CHWs. *Conclusions* The study results, combined with a scan of existing competencies, led to a tailored set of competencies that serve as the foundation for a HS CHW training program. This training program has the capacity to advance strategic goals for HS by strengthening HS CHWs’ capacity nationwide to respond to complex participant needs. Other maternal and child health programs may find these results of interest as they consider how CHWs could be used to strengthen service delivery.

## Significance

Peer reviewed literature has demonstrated that CHWs can positively impact many maternal and child health outcomes, including infant birth weight. This study provides a unique look at how Healthy Start (HS) community health workers (CHW), as critical members of the workforce, currently serve families and communities. Study results led to a tailored set of competencies that serve as the foundation for a HS CHW training program. Other perinatal health programs may find these results, as well as the process, of interest as they consider how CHWs could be used to strengthen their own service delivery.

## Introduction

Birth weight is an important predictor of short-and long-term infant health outcomes (Matthews et al. 2015; Fanaroff et al. 2007; Malin et al. 2014). Despite improvements in birth outcomes over the last 20 years, disparities persist (Martin et al. 2017). The percentage of infants born low birthweight increased among births to non-Hispanic black (13.2–13.4%) and Hispanic (7.1–7.2%) mothers while the low birth weight level for infants born to non-Hispanic white women remained stable (6.9%). Additionally, infants born preterm have higher infant mortality rates than full-term infants (Matthews et al. 2015). Similarly, the preterm birth rate increased slightly from 2014 to 2015 due to increases in the preterm birth rates for infants born to non-Hispanic black (13.2–13.4%) and Hispanic (9.0–9.1%) women, while the rate for infants born to non-Hispanic white women remained stable (8.9%).

The Healthy Start (HS) program is dedicated to preventing infant mortality, improving birth outcomes, and reducing disparities in maternal and infant health. HS began in 1991 as a demonstration project that funded 15 local sites. The U.S. Department of Health and Human Services (HHS) Health Resources and Services Administration (HRSA) currently funds 100 local HS programs across 37 states and Washington, DC. Past evaluations of HS noted variability across local programs and emphasized the importance of further ingraining social determinants of health principles into the program model (Kotelchuck 2010). Recommendations to increase the consistency with which services are delivered and to further address social determinants of health were incorporated into the reenvisioned HS program and reinforced the HRSA Strategic Goals to (1) Improve Access to Quality Health Care and Services, (2) Strengthen the Health Workforce, (3) Build Healthy Communities, and (4) Improve Health Equity (HRSA 2014).

HS uses a community-based participatory approach to provide culturally competent, family-centered, and comprehensive health and social services to women, infants, and their families. Community health workers (CHW) are essential to this service delivery model, and have been crucial members of the HS workforce since the initiative’s inception (Simon and Reykovich 1994). They are frontline public health workers who are trusted members and/or have a unique understanding of the community served (APHA). Peer reviewed literature has demonstrated that CHW can positively impact many maternal and child health outcomes, including infant birth weight (Redding et al. 2015; Rotheram-Borus et al. 2014). Similar to CHWs in other programs (HRSA 2007), HS CHWs work to reduce ethnic and racial disparities in maternal and infant health, recognizing that the root causes of these disparities include the social determinants of health—the conditions in which people are born, grow, live, work and age—as well as differences in access to health care (Healthy People 2020).

To support HRSA’s goals of a reenvisioned HS program with a workforce better equipped to manage complex participant needs, the HS EPIC Center, the training and technical assistance center for HS grantees, sought to create a standardized training program for CHWs. This study explored how CHWs serve HS participants, what roles they take on, and what skills they need to have, in order to inform the development of the standardized training program.

## Methods

Methods included an online questionnaire for program directors/managers and discussion groups with CHWs and CHW trainers/supervisors. The survey was reviewed and found exempt by the JSI Institutional Review Board. Discussion groups participants provided informed verbal consent before the session.

The online questionnaire was developed by one of the authors. The questionnaire examined the following topics: overview of CHW program, roles, training, skills, and perceived impact of CHWs on HS program outcomes. The survey questions and response options, including the list of roles and skills, were informed by a review of the literature on existing CHW programs with defined core competencies, and then vetted and refined by five initial volunteer HS grantee interviewees. The questionnaire defined CHWs as follows: “Individuals whose duties include at least some of the following: outreach, health coaching, health education, home visitation, care coordination, helping with navigation, patient advocacy and/or community engagement. CHWs generally do not hold a professional license or provide clinical care.” Respondents were also asked to consider both directly employed and subcontracted CHWs.

The questionnaire, programmed in Survey Gizmo, was sent to program directors/managers in all 100 HS programs in July 2015. Support from federal program leadership for the study yielded a very high response rate (93%) by August 31, 2015. Though there is heterogeneity among HS sites, the response rate of nearly all sites enabled the authors to draw conclusions about CHWs in the program without requiring extrapolations. Frequencies were calculated using SPSS Statistics.

Following the survey, five discussion groups were conducted, three with HS CHWs (n = 21) and two with CHW trainers/supervisors (n = 14) in October 2015. The purpose of these discussion groups was to further examine the knowledge, skills, and training HS CHWs require and to inform the development of a standardized training program for HS CHWs. CHWs in the discussion groups represented six HS programs, one in California and five in Michigan. Trainers/supervisors represented three HS programs and external trainers from non-HS organizations. CHWs represented a variety of job titles including advocate, outreach worker, and patient navigator. CHWs received a gift card as a thank you for their participation.

Each discussion group lasted 90 min and followed a standardized discussion guide. The discussion guide was organized around knowledge and skills related to (1) preconception health, prenatal health, postpartum health, and early childhood health and development (collectively referred to as the four perinatal periods for HS^1^), and (2) job responsibilities that were most frequently identified by survey respondents as essential (see “Results” section): outreach and recruitment, connecting participants to services, health education, and health coaching. Discussion group sessions were recorded with the permission of participants. Recordings were then transcribed and two of the authors manually analyzed the transcriptions for themes within the four perinatal periods and four job responsibilities discussed above. Once themes were identified, the two reviewers compared findings to ensure agreement.

## Results

### Organizational Survey Results

Out of 100 programs, 93 HS managers/directors responded to the survey. Overall 91% of respondents reported that their HS program used CHWs in some capacity. Approximately one-third (29%) of respondents whose programs use CHWs reported that their HS program subcontracts for some or all CHW services.

### Characteristics

**Fig. 1:**
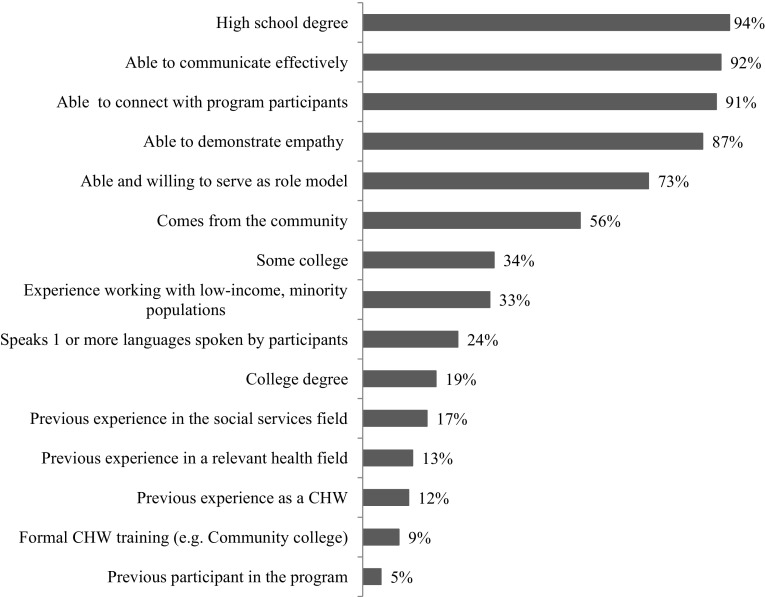
Percent of organizational survey respondents indicating characteristic is essential for CHWs to perform well in the Healthy Start program (n = 86)

Survey respondents were asked to rank the degree to which a number of characteristics were important for the CHW to perform well in their job serving clients on a four-point scale: essential, very useful, useful, and not necessary. Four of the top five most critical characteristics were personality traits: ability to communicate, ability to connect, demonstrate empathy, and serve as role model (Fig. 1). “Coming from the community”, often used to define CHWs, was the sixth most important characteristic of CHWs according to respondents. Few respondents (9%) rated formal CHW training an essential characteristic.

### Training

**Fig. 2:**
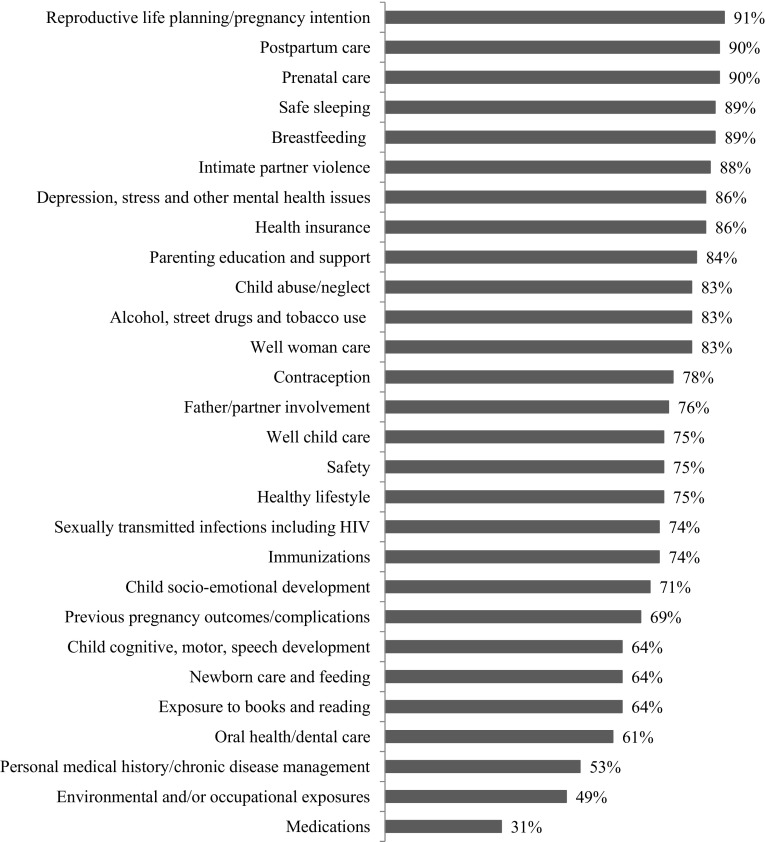
Percent of organizational survey respondents indicating health-related topic is covered in CHW initial training (n = 80)

There was large variation in the amount and content of training provided to CHWs in HS. While 89% of respondents reported providing training to hired CHWs at entry, 68% reported that subcontracted CHWs receive entry training, and half of respondents (50%) reported that hired and subcontracted CHWs receive different entry training. The amount of training varied across programs: from less than 10 h (5%), 11–40 h (35%), 41–80 h (32%), and more than 80 h (29%). Survey respondents were asked to review a list of health-related topics and indicate which were included in their entry CHW training. The list of topics included the HS performance measures, as well as topics deemed important to HS by the initial interviewees. There was substantial variation in what topics are covered in training, with a range of 31–91% across the 28 topics (Fig. 2).

### Roles

**Fig. 3:**
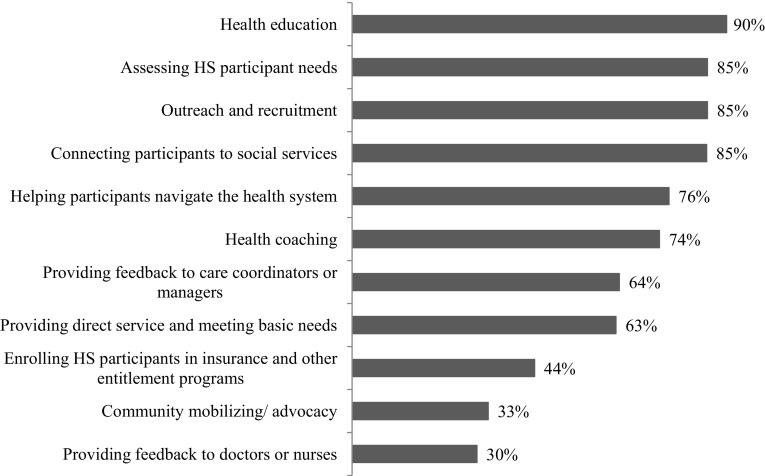
Percent of organizational survey respondents indicating activity is central to CHWs role in the Healthy Start program (n = 87)

Survey respondents were asked to rank the degree to which a number of activities were central to the role/job responsibilities of their CHWs on a four-point scale: activity is central to the job, not central but an important task, occasional involvement, and does not engage in this activity. Health education (defined as providing basic information to participants regarding healthy practices), assessing participant needs, participant recruitment, and connecting participants to social services were viewed as the four most important activities for HS CHWs with over 80% of respondents agreeing these activities are central to the CHW’s role (Fig. 3). Less than a third (30%) of respondents viewed providing nurses and doctors with feedback as central to the CHW role in HS.

### Skills

**Fig. 4:**
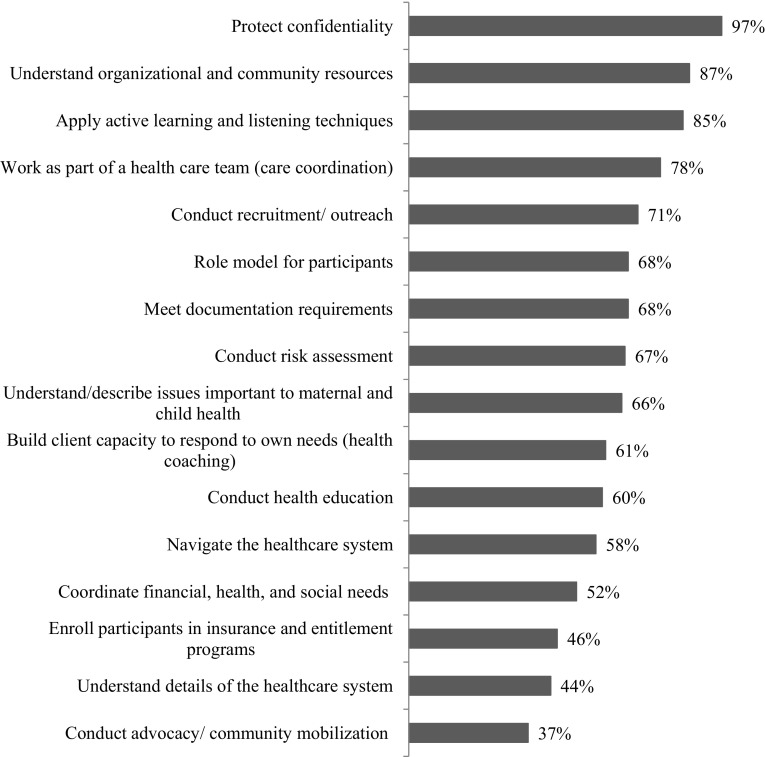
Percent of organizational survey respondents indicating skill is essential to CHWs role in the Healthy Start program (n = 88)

Survey respondents were asked to rank the degree of importance for a number of skills on a four-point scale: essential, very useful, useful, and not necessary. The top three ranked skills, with over 80% of respondents rating each “essential”, were protecting confidentiality, understanding organizational and community resources, and applying active learning and listening techniques (Fig. 4). Skills least often rated “essential” were enrolling participants in insurance and entitlement programs, understanding details of the healthcare system, and conducting advocacy/community mobilization.

### Perceived Impact

**Fig. 5:**
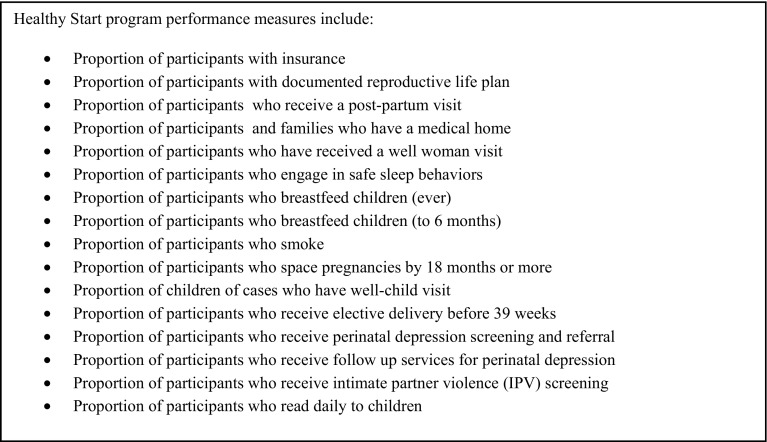
Healthy start program performance measures

Program directors/managers were then asked to determine whether CHWs in their program could positively impact each of 16 HS program performance measures (Fig. 5). Over 75% of respondents reported that CHWs could positively impact results on 15 out of 16 HS performance measures. The only performance measure which fewer than 75% of respondents indicated CHWs could positively influence outcomes was the proportion of participants who receive elective delivery before 39 weeks (60%).

### Discussion Group Results

Discussion groups explored: (1) general knowledge and skills needed by HS CHWs (2) knowledge and skills specific to the roles of outreach and recruitment, connecting participants to services, and health education; and (3) challenges HS CHWs face in their work. These specific areas were chosen based on the organizational survey findings. Within each of these areas, themes regarding knowledge and skills necessary to perform as a HS CHW emerged. Illustrative comments are included.

### General

CHWs and trainers identified several general knowledge and skills domains. First, they highlighted how important it is for CHWs to know the community and services available. The ability to develop strong relationships with participants was also emphasized. Essential interpersonal skills included responsiveness to participant needs, ability to build trusting relationships, provision of peer support, and ability to build client capacity to advocate for themselves and their families.

- “It’s really important for us to build that connection or a relationship with them so they can trust in us…so we can continue working during the year or 2 years.” —CHW
- “I like the fact that I can empower women who need to be empowered and they need to know that they can do it.” —CHW
- “Our role [is] helping to unleash [the CHWs’] intrinsic strengths and qualities and skills. Inspire them to adopt the leadership. To be advocates on behalf of themselves and their communities and people that they can help.” —Supervisor/trainer

### Outreach and Recruitment

CHWs and supervisors/trainers agreed that in order to fulfill the core CHW role of outreach and participant recruitment, CHWs must be able to map potential outreach locations; visit clinics and community-based organizations to educate staff about HS; effectively communicate information about the program throughout the community; exchange information with potential participants; develop referral networks; and encourage participants and community members to spread the word about HS services available.

- “I just go and say, ‘Hi my name is [hidden], I work with Healthy Start, and we’re a national program. We want you to have a healthy pregnancy, a healthy baby, and a healthy life. We have community resources that we can assist you with.” —CHW
- “In terms of outreach, we also have an asset map and I found that really, basically it’s my guide…It has been very useful in terms of creating partnerships with other organizations.” —CHW

### Connecting Participants to Services

To fulfill the role of connecting participants to services, CHWs and supervisors/trainers identified the following knowledge and skills as especially important. First, CHWs must be adept in making a wide variety of referrals to meet complex participant needs, including medical services, social services, substance abuse counseling/treatment, and community programs. Second, CHWs must be able to motivate participants to problem solve and build participants’ own capacity to access these community resources.

- “Our job is to find these women, be sure that they’re seeing a doctor, be sure that they’re getting WIC, [and] be sure that we have other resources that they need.” —CHW
- “How do you appropriately do a warm hand-off referral? How do you document your referrals? How do you follow up on your referrals? All those things…make a difference in being what I always like to say, a mediocre worker or an excellent worker.” —Supervisor/trainer

### Health Education

Certain knowledge is essential for HS CHWs to have given the nature of the program serving women, infants, and families. This knowledge includes the multiple health topics appropriate to the four HS perinatal periods (i.e., preconception, prenatal, postpartum, and parenting), as well as child developmental stages. CHWs must be able to effectively communicate this information (e.g., taking into account participant reading level). Finally, CHWs should be able to effectively encourage participation in group health education.

- “We encourage breastfeeding and just the knowledge of formula versus breast milk and re-educating on that. I guess milestones, development, and what they should be doing, how to soothe, and to put up with crying.” —CHW
- “Every visit that we go with, we are prepared with a key message. At the first visit we talk to them about the importance of going to the doctor for prenatal care, the vitamins, and so on. If they want to know about a specific topic yeah of course we’ll bring that information or we connect them to the agency or clinic where they can provide an assist to give more information, counseling [and] training about that.” —CHW

### Challenges

CHWs and supervisors/trainers identified a number of challenges CHWs face. Many related to working with participants who have complex and often overwhelming social needs that impact their health (e.g., unstable housing, lack of childcare, food insecurity). Other challenges included loss of participants to follow up, participant mistrust of social service programs, personal safety of CHWs on the job, shrinking social service system networks, and gaps in services offered.

- “I come into contact with different clients and they have so many issues, so many, they are burdened down by so many things.” —CHW
- “Sometimes for us as navigators, the big challenges that we have, I would say 99% of my program participants come to me and ‘I’m looking for low income housing.’” —CHW

## Discussion

### Roles and Skills

Survey findings indicate the majority of HS CHWs conduct outreach and recruitment, assess client needs and link them to health and social services, and provide health education. To conduct these roles, CHWs must have a number of skills. They must have an understanding of the wide range of maternal and infant health issues. They must be skilled at meeting potential participants where they are and persuading them of the value of the HS program. Additionally, they need to have the ability to connect clients to services appropriately, which involves a very deep understanding of community assets. Strong communication skills are essential for CHWs to meet complex participant needs with compassion. This is reflected in the emphasis program directors/managers placed on communication skills when considering characteristics in the hiring process.

Although there was general agreement in key roles of HS CHWs, and how these relate to addressing social determinants of health, one area where there was a lesser degree of alignment was how CHWs are integrated into medical care teams. This likely reflects the fact that a minority of HS programs are embedded in clinical settings of health centers and hospitals (17%) (Healthy Start EPIC Center 2015). CHWs in non-clinical settings may be more likely to interact with HS case managers rather than directly with clinical providers.

### Training

Study findings showed wide variation in the training provided to CHWs in HS programs. Given the general agreement among respondents regarding the roles of CHWs, the variability in training highlighted an opportunity to enhance training to support these roles. In particular, with health education being a core role, it is important that HS CHWs have a common understanding of the leading maternal and infant health issues. Linking women, infants, and families to services throughout the four HS perinatal periods is a skill to be developed on the local level, as CHWs need to understand existing community assets. Importantly, the results identified an opportunity to strengthen the HS workforce by building CHW skills in the areas of outreach, needs assessment and service navigation, as well as increasing the consistency with which those skills are applied. These findings reiterated the utility of a standardized training program that strengthens these identified skills. There was general consensus among survey respondents that CHWs can positively impact HS performance measures, indicating that building the capacity of CHWs through training is a worthwhile investment.

### Implications for Workforce Development

The survey and discussion group results provided a detailed understanding of the essential roles, necessary skills and daily challenges of HS CHWs, and enabled the HS program to identify broad areas of competency needed to effectively serve HS participants, a first step toward developing a standardized training program. A scan of the literature identified core competencies common to many CHW training programs, including: outreach methods and strategies, individual and community assessment, effective communication, cultural responsiveness, health education, care coordination and system navigation (CDC 2014; CHW Net NYC, NYHF, and CUMSPH 2011; Dickson and Yahna 2012; MA Board of Certification of CHWs 2014; MN CHW Curriculum 2013). Our findings, which also highlighted the importance of outreach, health education, care coordination, assessment, and communication, align strongly with these broadly accepted competencies.

**Fig. 6:**
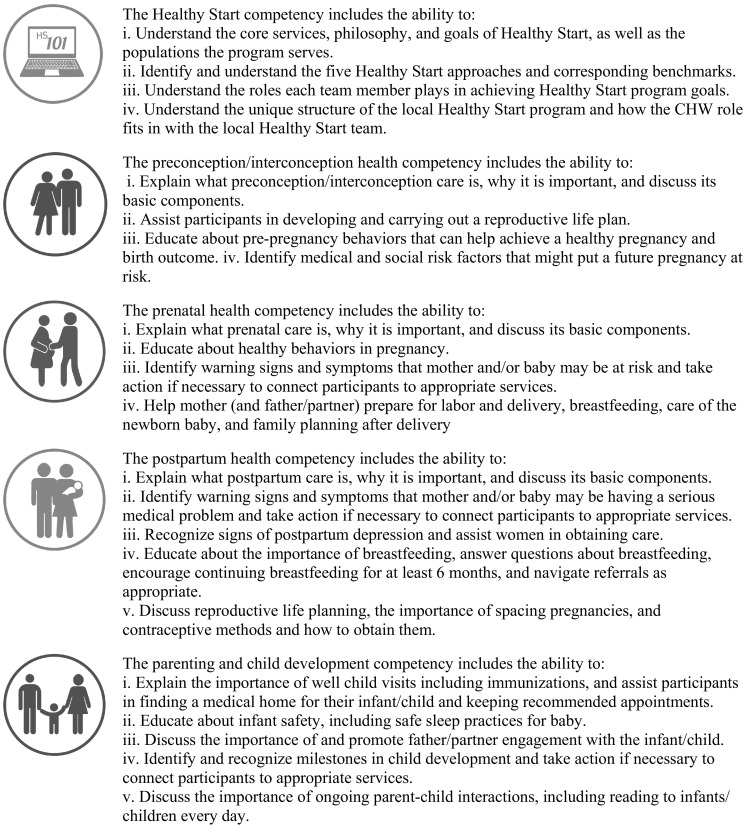
Healthy Start and perinatal health competencies

The HS EPIC Center selected the Massachusetts Board of Certification of Community Health Workers’ Core Competencies (MA Board of Certification of CHWs 2014) as competencies on which to build the training program, as they were comprehensive and reflective of consensus in the field. These competencies were then tailored to reflect CHW scope of work within the HS program based on survey findings and discussion groups. Five knowledge-oriented competencies were added including a HS competency, and competencies corresponding to the four HS perinatal periods. The draft competencies were reviewed and revised using a consensus process, by a CHW Course Development Advisory Group, composed of HS grantees, a HS CHW, and HRSA representatives. The five HS-specific competencies are found in Fig. 6.^2^

These core competencies represent the fundamental knowledge, experience, and skills needed to fulfill the roles and responsibilities of a HS CHW, and effectively serve HS participants. Using these competencies as a foundation, the HS EPIC Center developed a multi-module online training program that is available for free to all HS programs. HS programs are encouraged to consider how the modules complement existing training that they provide to CHWs. Although not required, it is strongly recommended all HS CHWs participate in the training program as part of their own professional development, and to help ensure HS CHWs nationwide share a common foundation of basic knowledge and skills needed to effectively serve the complex needs of HS participants.

### Limitations

Although a very high response rate of 93% was achieved for the organizational survey, a limitation of this study is that discussion groups were limited to six HS sites in two states (CA and MI) who volunteered to participate. Thus, the results of the discussion groups are not generalizable to all 100 HS grantees. It is important to examine the discussion group results as elucidatory of, and not separate from, the organizational CHW program survey. Second, this analysis was focused on one federal program and may limit the generalizability of results to other perinatal health programs, as they may use CHWs differently.

## Conclusions

This study provided a unique opportunity to fully examine the CHW landscape within one federal maternal and infant health program, HS. It can be concluded that HS CHWs’ primary roles include conducting outreach and recruitment, providing health education, and linking clients to appropriate services, addressing a broad spectrum of needs across the social determinants of health. In conducting these roles, CHWs must be responsive to and address underlying social determinants of health. When the HS program was reenvisioned in 2014, priorities included standardization of services and strengthening of the HS workforce to more effectively meet complex participant needs. The survey and discussion groups findings, combined with a scan of existing competencies, were used to a tailor a set of competencies that serve as the foundation for a HS CHW training program. This program has the potential to enhance the ability of the HS workforce to effectively serve participants, and advance the strategic goals for the HS program. Other maternal and child health programs may find these results of interest as they consider how CHWs are and could be used to strengthen their own service delivery.

## Footnotes

1 HS programs focus on serving women and families from preconception through pregnancy, and up until the child’s second birthday.

2 The full competencies are available on the EPIC Center website: healthystartepic.org/training-and-events/community-health-worker-survey/.
